# Ambulatory bilateral groin hernia repair: open preperitoneal versus laparoscopic outcomes

**DOI:** 10.1007/s10029-025-03523-4

**Published:** 2025-11-24

**Authors:** Maria Jose Gomez-Jurado, Mireia Verdaguer-Tremolosa, Victor Rodrigues-Gonçalves, Pilar Martínez-López, María Martínez-López, Meritxell Pera, Mar Dalmau, Manuel López-Cano

**Affiliations:** 1 General and Digestive Surgery Department, Dr. Josep Trueta University Hospital, Girona, Spain; 2 General and Digestive Surgery Department, Vall d’Hebron University Hospital, Barcelona, Spain; 3 Department of Surgery, Universitat Autònoma de Barcelona, Barcelona, Spain; 4 Abdominal Wall Unit, General and Digestive Surgery Department, Vall d’Hebron University Hospital, Barcelona, Spain

**Keywords:** Bilateral groin hernia, Open preperitoneal hernia repair, Transabdominal preperitoneal inguinal repair, Totally extraperitoneal inguinal repair, Chronic postoperative inguinal pain

## Abstract

**Purpose:**

To evaluate short and long-term outcomes of bilateral groin hernia (BGH) repair using an open preperitoneal approach (OPA) compared to minimally invasive surgery (MIS) in ambulatory surgery.

**Methods:**

A retrospective cohort study was conducted including patients undergoing ambulatory BGH repair between 2010 and 2018 at Vall d’Hebron University Hospital (Barcelona) using either OPA (a modified Wantz technique) or MIS [transabdominal preperitoneal (TAPP) or totally extraperitoneal (TEP)]. Demographic, perioperative, and postoperative data were analysed. Chronic postoperative inguinal pain (CPIP) was assessed at two time points: early (3–12 months) and late (> 12 months postoperatively). Long-term follow-up was conducted through structured telephone interviews using the Hernia Recurrence Inventory survey. Multivariate logistic regression and ROC analysis were used to identify predictors of CPIP.

**Results:**

A total of 244 patients (488 hernias) met the inclusion criteria, with a median follow-up of 116 months. OPA patients were older and had more comorbidities (*P* < 0.001). Operative time was shorter in the OPA group (median 70 vs. 110 min; *P* < 0.001). No significant differences were found in recurrence rates or surgical site occurrences. Multivariate analysis showed that OPA was independently associated with a lower risk of CPIP between 3–12 months postoperatively (OR 0.091, *P* < 0.001) compared to MIS. At long-term follow-up, higher Body Mass Index (BMI) was the only factor associated with persistent pain (OR 1.2, *P* = 0.024).

**Conclusion:**

OPA is a safe and effective technique for BGH repair, offering shorter operative times and lower risk of CPIP between 3–12 months postoperatively compared to MIS, while maintaining comparable long-term outcomes (> 12 months).

## Introduction

Over the years, multiple surgical techniques have been developed for inguinal hernia repair, each offering specific advantages and limitations. The ideal technique should be reproducible, cost-effective, and associated with low recurrence and chronic pain rates, minimal complications, and a fast, safe recovery [1].

The HerniaSurge group published the first international guidelines for the management of bilateral groin hernia (BGH), recommending minimally invasive surgery (MIS)—either transabdominal preperitoneal (TAPP) or totally extraperitoneal (TEP) —as the preferred approach for primary BGH, with prosthetic mesh placement in the preperitoneal space [2]. However, the guidelines and other authors [3, 4] also emphasize that the choice of approach may depend on the surgeon’s expertise in either open or endoscopic techniques. In such cases, an open anterior approach, such as Lichtenstein repair, is proposed as an alternative. This recommendation is partly based on the fact that Lichtenstein remains the most widely used technique worldwide for groin hernia repair among surgeons from different hernia societies [5].

Unlike anterior mesh placement, preperitoneal mesh positioning has been proposed to benefit from certain biomechanical advantages. By covering the myopectineal orifice of Fruchaud and being supported by intra-abdominal pressure (‘upstream principle’), it may allow broader reinforcement with potentially less need for fixation [6, 7] This setting reduces the need for traumatic fixation methods, which are more frequently required in anterior mesh repairs and may increase the risk of nerve entrapment and chronic postoperative inguinal pain (CPIP) [6, 8–10]. Additionally, preperitoneal mesh placement provides simultaneous coverage of the femoral canal, thereby reducing the risk of occult femoral hernias—an advantage particularly relevant in female patients, who show a higher rate of femoral recurrences after anterior repairs [2].

Previous studies comparing open preperitoneal approaches (OPA) to MIS have shown similar outcomes regarding chronic pain, complications, and recurrence rates [11, 12]. However, most of this evidence comes from studies including unilateral cases or mixed techniques. To date, there is a lack of specific comparative data focused exclusively on pure OPA versus MIS for the repair of BGH.

This study aims to compare OPA and MIS (TAPP and TEP) in the surgical treatment of BGH in an ambulatory setting. We present the results of a single-centre experience with long-term follow-up, focusing on clinical outcomes such as operative time, complications, recurrence, and CPIP. Our prespecified hypothesis was that OPA would achieve outcomes comparable to MIS in selected patients.

## Patients and methods

This retrospective study included all patients with a BGH who underwent elective surgery using OPA or MIS in ambulatory surgery at the Abdominal Wall Surgery Unit of the Vall d'Hebron University Hospital in Barcelona (Spain) between January 2010 and September 2018, defining ambulatory surgery as surgery that does not require an overnight hospital stay [13]. The Abdominal Wall Surgery Unit is an accredited unit by the Spanish Association of Surgeons (Asociación Española de Cirujanos, AEC) [14].

The study was approved by the hospital ethics committee (PR(AG)505/2021) and informed consent for the intervention was obtained for all patients. Patients meeting the inclusion criteria for outpatient surgery were included: age over 18 years, primary and/or recurrent, inguinal or femoral bilateral hernia, American Society of Anaesthesiologists (ASA) grade I-III, non-obese patients [i.e. Body Mass Index (BMI) < 30], good exercise tolerance, well-controlled diabetes, well-controlled chronic obstructive pulmonary disease (COPD) or other comorbidities (i.e. hypertension, well-controlled ischaemic heart disease). Excluded from the data collected were the emergency repairs and the patients with poorly controlled comorbidities (non-ischemic or poorly controlled ischemic heart disease, stroke), use of anticoagulants and those classified like scrotal hernias. Additionally, patients who did not complete in-person visits or long-term follow-up through the telephone call were excluded from this study.

All patients were operated by the same surgeon who has an optimal prior learning curve of more than 100 repairs with the different procedures [2]. In our institution, open preperitoneal repair is systematically preferred over anterior open approaches for elective inguinal and femoral hernias, with the anterior approach reserved only for cases where the posterior plane has been previously altered.

This observational study has been designed and reported following the guidelines established by the STROBE [15] and RECORD [16] checklists to ensure the quality and transparency in the presentation of the results.

### Data collected

The collected data included demographic variables such as age, sex, BMI and smoking status. Comorbidities were recorded including diabetes, COPD and other relevant conditions (e.g., hypertension, well-controlled ischaemic heart disease) and ASA classification. Hernias were classified according to the European Hernia Society (EHS) classification [17] for both right and left sides, and cases of recurrence after previous repair were noted.

Operative data included the type of anaesthesia (spinal/general), method of mesh fixation (absorbable, non-absorbable, suture, tackers, or no fixation), intraoperative nerve or visceral injuries, conversion from MIS to open surgery, and duration of surgery (in minutes), measured from skin incision to placement of the final dressing.

Postoperative complications during the first 30 days included superficial and deep wound infections (classified according to Centers for Disease Control and Prevention [CDC] criterio) [18], as well as hematomas—defined as any visible accumulation of blood under the skin- and seromas. All of these were categorised as surgical site occurrences (SSO) [19].

We recorded hernia recurrence, clinically assessed and classified as unilateral or bilateral, and CPIP, defined according to the HerniaSurge guidelines as “postoperative inguinal pain including a level of discomfort rated by the patient as at least ‘moderate’, impacting daily activities, and lasting longer than three months” [1]. Both variables were recorded as present or absent.

CPIP recorded between 3 and 12 months postoperatively during scheduled follow-up visits was classified as 'early CPIP', to differentiate it from pain persisting beyond 12 months. This distinction was made in line with previous studies suggesting a progressive decline in pain over time, with persistent pain becoming less common after the first postoperative year [20–24]. Pain was assessed using a four-point categorical scale (none, mild, moderate, severe), a method previously validated in studies evaluating postoperative pain in hernia surgery [25, 26]. Mild pain was defined as occasional discomfort not interfering with daily activities after returning to pre-hernia lifestyle and not requiring analgesics. Moderate pain referred to discomfort that interfered with daily activities, though analgesic use was rare. Severe pain was defined as pain that frequently impaired daily functioning or was disabling, often requiring the use of pain medication.

Follow-up at the latest available time point (beyond 12 months postoperatively) was performed through a telephone interview using the hernia recurrence inventory survey proposed by Tastaldi et al. [27], consisting of three questions: Q1. Do you think the hernia has come back? Q2. Do you feel or see a bulge? Q3. Do you have physical pain or symptoms at the site?

Finally, mortality within the first 30 postoperative days was also recorded.

### Surgical techniques

Surgical approach decision was left to surgeon discretion based on a combination of patient-specific factors like patient's surgical history, suspicion or BGH confirmed and the available resources in our clinical setting.

#### OPA

The OPA was performed separately for each hernia using a modified Wantz technique [28], under either spinal or general anaesthesia, depending on the anaesthesiologist’s assessment. A horizontal lower abdominal incision was made cranial to the inguinal ligament and above the internal ring, with dissection carried through the tissue layers to access the preperitoneal space. Dissection and hernia reduction were performed under direct vision of the myopectineal orifice.

A 15 × 15 cm wide-pore, medium-density polypropylene mesh was placed in the preperitoneal space and fixed with a single absorbable monofilament suture (2/0) to pectineal ligament. A lateral slit was made in the mesh to create two tails, through which the spermatic cord or round ligament was passed. The upper tail was crossed over the lower and sutured together with an absorbable monofilament stitch (2/0), then tucked beneath the transversalis fascia. The mesh extended beyond the limits of the myopectineal orifice to cover all potential hernia defects. Closure was performed in layers using absorbable monofilament sutures [29]. Skin closure was subcuticular, using either absorbable or non-absorbable monofilament, depending on surgeon preference.

#### MIS

The minimally invasive techniques included TAPP and TEP approaches, both performed under general anaesthesia. Procedures followed standard principles [30, 31], with minor adaptations.

For TAPP, a supraumbilical Hasson trocar was placed, and pneumoperitoneum was established. Two additional 5 mm trocars were inserted on each side of the Hasson and laterally to the inferior epigastric vessels. The peritoneum was opened bilaterally above the hernia defect. In female patients, the round ligament was transected at least 1 cm proximal to the internal ring to avoid injury to the genital branch of the genitofemoral nerve, which is often adherent to the peritoneum. Following complete dissection and exposure of the myopectineal orifice, each side was covered with a 15 × 15 cm wide-pore, medium-density polypropylene mesh. Mesh fixation was performed using permanent or absorbable tacks at the pectineal ligament, or with surgical glue depending on availability [31, 32]. The peritoneum was then closed with continuous absorbable sutures, ensuring full mesh coverage.

For TEP, the Hasson and two 5 mm trocars were placed in the midline below the umbilicus. The preperitoneal space was dissected bluntly via the infraumbilical trocar. Both inguinal regions were dissected with full visualization of the myopectineal orifices. As in TAPP, two 15 × 15 cm polypropylene meshes were placed to cover each side and fixed using tacks (permanent or absorbable) or glue, depending on resource availability and intraoperative judgement. The Hasson port was closed with absorbable material and the skin with monofilament sutures. Maximum CO₂ insufflation pressure was limited to 12 mmHg.

### Follow-up

According to our institutional protocol, all patients are routinely scheduled for in-person follow-up visits at 1, 6, and 12 months postoperatively, aimed at detecting early complications, addressing persistent pain, and confirming absence of recurrence before discharge. Subsequently, as part of the study protocol, a structured telephone interview was conducted to assess for hernia recurrence and CPIP using the previously mentioned Hernia Recurrence Inventory survey[27] and the four-point categorical scale of pain (none, mild, moderate, severe) respectively. Patients reporting symptoms, bulging, or uncertainty were systematically invited for in-person clinical evaluation to confirm or rule out recurrence.

### Statistical analysis

A comparison of the distribution of variables between patients with different surgical approaches (OPA and MIS) was made. Categorical variables are expressed as frequencies and percentages. Continuous variables are expressed as mean ± standard deviation (SD) if normally distributed, or median and interquartile range (IQR) otherwise. The chi-square test or the Fisher’s exact test were used for the comparison of categorical variables, and the Kruskal–Wallis test or the Mann–Whitney U test for the comparison of quantitative variables according to conditions of application. We performed logistic regression for multivariate analysis. Statistical significance was set at *P* < 0.05. The inclusion of the variables in the model was based on their significance in the univariate analysis (*P* < 0.05) and on clinical consensus. Postoperative complication rates are reported as odds ratios (ORs) with 95% confidence intervals (CIs). A multivariate logistic regression analysis was performed to adjust for potential confounders, and receiver operating characteristic (ROC) curves were constructed to assess the discriminatory ability of the model. The Statistical Package for the Social Sciences (SPSS) version 23.0 (IBM Statistics, Chicago, IL, USA) was used for the analysis of data.

## Results

**Fig. 1:**
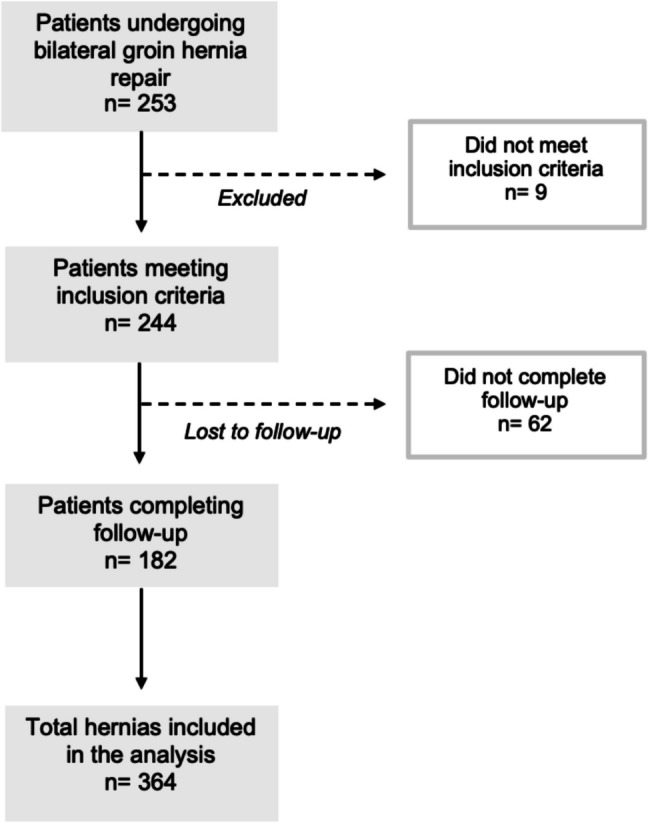
Study flow diagram showing patient and hernia inclusion, exclusions, and follow-up completion

During the study period, 253 consecutive patients underwent bilateral inguinal hernia repair, accounting for a total of 506 hernias. Of these, 244 patients (488 hernias) met the inclusion criteria and completed initial follow-up. Among them, 182 patients (364 hernias) reached long-term follow-up through telephone interviews (Fig. 1).

A total of 116 patients (64%) representing 232 hernia repairs underwent OPA, while 66 patients (36%) representing 132 hernia repairs underwent MIS, either TAPP or TEP technique, as detailed in Table 1.

**Table 1:** Demographic and surgical data per patient

Demographic data	Total (*n* = 182)	OPA (*n* = 116)	MIS (*n* = 66)	*P-*value
Years, median (IQR)	52 (43–61)	57 (48–63)	46 (41–53)	< 0.001
Male/female, (%)	170 (93)/12 (7)	107 (92)/9 (8)	63 (95)/3 (5)	0.235
BMI, median (IQR)	25 (24–28)	26 (24–28)	25 (23–27)	0.48
Smoker (%)	55 (30)	30 (26)	25 (38)	0.016
Diabetes (%)	15 (8)	12 (10)	3 (5)	0.053
COPD (%)	4 (2)	4 (3)	0	0.055
Cardiovascular disease (%)	26 (14)	22 (19)	4 (6)	0.001
Previous inguinal surgery (%)	36 (20)	21 (18)	15 (23)	0.287
ASA I (%) ASA II (%) ASA III (%)	58 (32) 118 (55) 6 (3)	31 (27) 79 (68) 6 (5)	27 (41) 39 (59) 0	0.001

*BMI* body mass index, *COPD* chronic obstructive pulmonary disease, *ASA* American society of anaesthesiologists

### Patient, hernia and surgical characteristics

Patients in OPA group were significantly older (*P* < 0.001), with a minor proportion of smokers (*P* = 0.016) and more comorbidities such as cardiovascular disease (*P* = 0.001), leading to a minor proportion of ASA I classification compared to MIS group (*P* = 0.001) (Table 1). No other significant demographic differences were observed between groups.

Table 2 presents hernia characteristics and intraoperative data. Mesh fixation methods differed significantly between groups (*P* < 0.001): absorbable sutures were exclusively used in the OPA group, while non-absorbable tackers were the most frequent method in the MIS group (51%). The mean duration of surgery was significantly shorter in OPA group (median 70 min, IQR 60–75) than in the MIS group (mean 110 min, IQR 90–130; *P* < 0.001). There was also a significant difference in the anaesthesia technique used, with spinal anaesthesia preferred in OPA group (86%) and general anaesthesia used in 100% of MIS cases.

**Table 2:** Surgical data and postoperative events reported per hernia

Surgery data	Total (*n* = 364)	OPA (*n* = 232)	MIS (*n* = 132)	*P-*value
Recurrent hernia (%)	27 (7)	9 (5)	10 (12)	0.181
Right hernias (%)	182	116	66	0.375
Lateral	72 (39)	42 (36)	31 (47)	
Medial	103 (56)	68 (59)	34 (52)	
Femoral	7 (4)	6 (5)	1 (2)	
Left hernias (%)	182	116	66	0.330
Lateral	81 (44)	47 (40)	34 (52)	
Medial	96 (53)	66 (57)	30 (45)	
Femoral	5 (3)	3 (3)	2 (3)	
Mesh fixation (%)				
Non- absorbable tacker	68 (19)	0	68 (51)	< 0.001
Absorbable tacker	38 (10)	0	38 (29)	
Absorbable suture	234 (64)	232 (100)	2 (2)	
Non fixation	24 (7)	0	24 (18)	
Surgery time, median (IQR)*	75’ (65–105)	70’ (60–75)	110’ (90–130)	< 0.001
Anaesthesia (%)	182	116	66	< 0.001
General	82 (45)	16 (14)	66 (100)	
Regional	100 (55)	100 (86)	0	
EVENT (%)				
SSO	120 (33)	76 (33)	44 (33)	0.911
Recurrence	7 (2)	4 (2)	3 (2)	0.707
Early CPIP	66 (18)	8 (3)	58 (44)	< 0.001
CPIP at final follow-up	12 (3)	11 (5)	1 (1)	0.063

*OPA* open preperitoneal approach, *MIS* minimally invasive surgery, *SSO* surgical site occurrence, *CPIP* chronic postoperative inguinal pain

Two TEP cases were converted to TAPP due to accidental peritoneal breach during preperitoneal dissection. No conversions from MIS to OPA occurred. No nerve or visceral injuries were reported.

The median follow-up was 116 months (IQR 75–144, range 74–173). The laparoscopic group had a median follow-up of 119 months (IQR 83–141, range 74–173), similar to the OPA group [median 114 months (IQR 74–147, range 74–171); *P* = 0.67]. Of the 244 patients included, 182 (74.6%) completed long-term follow-up, while 62 (25.4%) were lost to follow-up over time, reflecting the challenges of maintaining surveillance in a cohort with a follow-up extending up to 14 years.

### Postoperative outcomes

Postoperative complications are shown in Table 2. No significant differences were observed in the overall rate of SSO (*P* = 0.911), with hematoma being the most common complication in both OPA (37%) and MIS (26%) groups (*P* = 0.713). Superficial wound infections occurred in 2 patients (4%) in the MIS group and 1 (2%) in the OPA group. All cases resolved with oral antibiotics and did not require drainage, corresponding to Clavien-Dindo grade II complications [33]. Given the very small number of cases, no meaningful statistical analysis could be performed, and these findings should be interpreted descriptively. There were no procedure-related deaths.

**Fig. 2:**
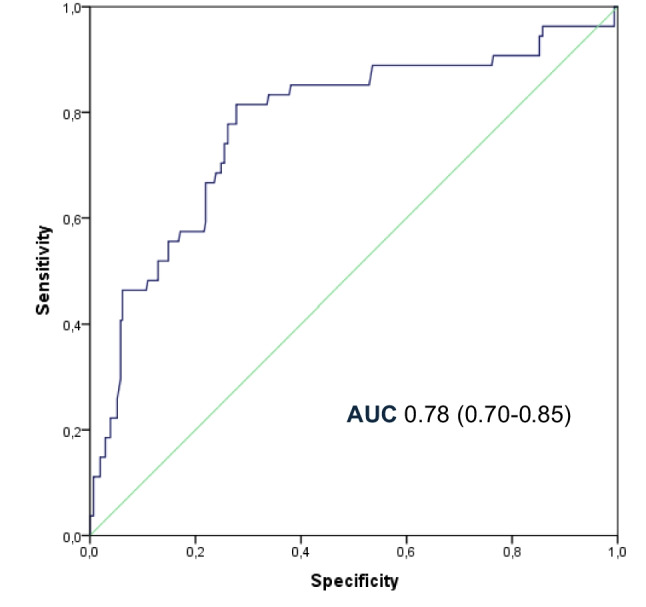
Receiver operating characteristic (ROC) curve for the logistic regression model predicting early CPIP

Early CPIP (3–12 months postoperatively) was more common in patients with recurrent hernias (*P* = 0.03), younger patients (*P* = 0.007) and those who underwent laparoscopic repair (*P* = 0.001) (Table 3). Absorbable suture fixation was associated with lower pain rates (*P* < 0.001). After adjustment for potential confounders, the final logistic regression model demonstrated good discriminative ability (AUC = 0.78; 95% CI: 0.70–0.85) (Fig. 2), with OPA showing a significant lower risk of early CPIP (OR = 0.091; 95% CI: 0.041–0.205; *P* < 0.001). No other independent risk factors associations were identified (Table 3).

**Table 3:** Univariate and multivariate analysis of early CPIP (pain reported after the third month)

Variables		Early CPIP (3–12 months PO)		Univariant analysis		Multivariant analysis	
		No *n* = 310	Yes *n* = 54	OR (95% CI)	*P*-value	OR (95% CI)	*P*-value
Recurrent hernia	No Yes	291 (94%) 19 (6%)	46 (85%) 8 (15%)	1 2.664 (1.102–6.439)	0.030	1 1.664 (0.606–4.566)	0.323
Previous inguinal surgery	No Yes	249 (80%) 61 (20%)	43 (80%) 11 (20%)	1 1.044 (0.509–2.143)	0.906		
Sex	Men Women	290 (93%) 20 (7%)	50 (93%) 4 (7%)	1 1.160 (0.381–3.536)	0.794		
Age		53 (43–61)	48 (41–55.5)	0.965 (0.940–0.990)	0.007	1.0020 (0.967–1.034)	0.982
BMI		25.4 (23.6–27.4)	26.7 (23.2–28)	1.034 (0.945–1.130)	0.470	1.062 (0.954–1.182)	0.271
Diabetes mellitus	No Yes	284 (92%) 26 (8%)	50 (93%) 4 (7%)	1 0.874 (0.292–2.611)	0.809		
COPD	No Yes	302 (97%) 8 (3%)	54 (100%) 0 (0%)	NA			
Cardiovascular disease	No Yes	264 (85%) 46 (15%)	48 (89%) 6 (11%)	1 0.717 (0.290–1.773)	0.472		
ASA classification	1 2 3	97 (31%) 201 (65%) 12 (4%)	19 (35%) 34 (63%) 1 (2%)	1 0.864 (0.469–1.592) 0.425 (0.052–3.469)	0.638 0.425		
Hernia type	Medial Lateral Medial and lateral Femoral	165 (53%) 134 (43%) 1 (1%) 10 (3%)	33 (61%) 18 (33%) 1 (2%) 2 (4%)	1 0.672 (0.362–1.246) 5.000 (0.305–81.968) 1.000 (0.209–4.776)	0.207 0.259 1.000		
Fixation method	None Absorbable suture Absorbable tacker Non-absorbable tacker	15 (5%) 222 (72%) 27 (9%) 46 (15%)	9 (17%) 12 (22%) 11 (20%) 22 (41%)	1 0.090 (0.033–0.247) 0.679 (0.230–2.007) 0.797 (0.302–2.103)	< 0.001 0.484 0.647		
Recurrence	No Yes	305 (98%) 5 (2%)	52 (96%) 2 (4%)	1 2.346 (0.443–12.413)	0.316		
SSO	No Yes	209 (67%) 101 (33%)	35 (65%) 19 (35%)	1 1.123 (0.612–2.061)	0.707		
Technique	Laparoscopy Open	88 (29%) 222 (72%)	44 (81%) 10 (18%)	1 0.090 (0.043–0.187)	< 0.001	1 0.091 (0.041–0.205)	< 0.001

### Long-term follow-up

All cases of pain reported during the long-term follow-up period (> 12 months postoperatively) were rated as mild during structured telephone interviews. Only two patients required temporary treatment with gabapentin, which was discontinued after full resolution of symptoms. No further interventions—such as nerve blocks or reoperations—were necessary. The only factor significantly associated with increased CPIP was a higher BMI (OR = 1.2, 95% CI: 1.024–1.405; *P* = 0.024) (Table 4). No significant differences in CPIP were found between surgical techniques (*P* = 0.07) or in relation to other demographic or clinical variables.

**Table 4:** Univariate and multivariate analysis of CPIP at maximum follow-up

Variables		Pain at long-term follow-up (> 12 months)		Univariate analysis		Multivariate analysis	
		No *n* = 52	Yes *n* = 12	OR (95% IC)	*P*-value	OR (95% IC)	*P*-value
Recurrent hernia	No Yes	325 (92%) 27 (8%)	12 (100%) 0 (0%)	NA			
Previous inguinal surgery	No Yes	282 (80%) 70 (20%)	10 (83%) 2 (17%)	1 0.806 (0.173–3.760)	0.783		
Sex	Men Women	329 (94%) 23 (7%)	11 (92%) 1 (8%)	1 1.30 (0.161–10.517)	0.805		
Age		54 (44–61)	47 (41–53.5)	0.996 (0.947–1.048)	0.890	0.977 (0.924–1.032)	0.401
BMI		25.3 (23.5–27.4)	26.4 (23.5–28.07)	1.239 (1.051–1.460)	0.011	1.200 (1.024–1.405)	0.024
Diabetes mellitus	No Yes	323 (92%) 29 (8%)	11 (92%) 1 (8%)	1 1.013 (0.126–8.122)	0.991		
COPD	No Yes	344 (98%) 8 (2%)	12 (100%) 0 (0,00%)	NA			
Cardiovascular disease	No Yes	300 (85%) 52 (15%)	12 (100%) 0 (0%)	NA			
ASA classification	1 2 3	112 (32%) 229 (65%) 11 (3%)	4 (33%) 7 (58%) 1 (8%)	1 0.856 (0.245–2.985) 2.545 (0.261–24.815)	0.807 0.421		
Hernia type	Medial Lateral Medial and lateral Femoral	190 (54%) 149 (42%) 2 (1%) 11 (3%)	8 (67%) 3 (25%) 0 (0%) 1 (8%)	NA			
Fixation method	None Absorbable suture Absorbable tacker Non-absorbable tacker	23 (7%) 223 (63%) 38 (11%) 68 (19%)	1 (8%) 11 (92%) 0 (0%) 0 (0%)	NA			
Recurrence	No Yes	346 (98%) 6 (2%)	11 (92%) 1 (8%)	1 5.242 (0.581–47.335)	0.140		
SSO	No Yes	235 (67%) 117 (33%)	9 (75%) 3 (25%)	1 0.670 (0.178–2.520)	0.553		
Technique	Laparoscopy Open	131 (37%) 221 (63%)	1 (8%) 11 (92%)	1 6.52 (0.832–51.081)	0.074	1 7.066 (0.852–58.596)	0.070
Early CPIP	No Si	289 (82%) 63 (18%)	9 (75%) 3 (25%)	1 1.529 (0.402–5.809)	0.533		

Hernia recurrence occurred in 4 cases (2%) in the OPA group and 3 cases (2%) in the MIS group, with no significant difference observed (*P* = 0.707) (Table 2).

## Discussion

In the context of international guidelines recommending a preperitoneal approach for BGH—typically via MIS—our study evaluates a pure OPA as a potential alternative. Our findings show that OPA achieves comparable outcomes to MIS in terms of recurrence and surgical site complications, with the added benefit of significantly shorter operative time. Notably, OPA was independently associated with a lower risk of early CPIP, suggesting a potential protective effect during the initial recovery phase. In contrast, higher BMI was the only variable significantly associated with persistent CPIP beyond 12 months.

Earlier studies suggested that laparoscopic repairs might reduce postoperative pain and infection rates [34]. However, a meta-analysis of available data found no significant differences between laparoscopic and open preperitoneal approaches regarding CPIP, recurrence, or local complications [11]. More recently, a comparison of robotic-assisted laparoscopic and open preperitoneal repairs likewise reported no significant differences in clinical outcomes, reinforcing the equivalence of these techniques [12].

Although we observed a lower incidence of CPIP between 3 and 12 months in the open preperitoneal group, some literature suggest that the absolute difference tends to decrease over time [20–24]. Long-term registry data and a randomized trial with long-term follow-up report a gradual resolution of pain in most patients regardless of surgical technique, with convergence in prevalence rates and quality-of-life measures over time [35–37]. Nevertheless, a lower incidence of early CPIP may represent a clinical advantage, potentially improving quality of life during the initial recovery phase and facilitating an earlier return to work and daily activities, although these outcomes were not specifically assessed in our study. Notably, in our study, early CPIP was not associated with pain at long-term follow-up, in contrast with previous studies that have identified it as a potential risk factor for long-term CPIP [24].

Importantly, most clinical guidelines base their recommendations in favour of MIS (TAPP or TEP) on comparisons with anterior open repairs, particularly the Lichtenstein technique [2]. However, there is a notable lack of data evaluating OPA in this context. Several studies and meta-analyses have shown that posterior mesh placement—whether via MIS or OPA—is associated with less acute and chronic pain, fewer surgical site occurrences, and better quality of life compared to anterior repairs, with similar recurrence rates [9, 11, 38–41]. In addition, the preperitoneal position of the mesh provides coverage of both the inguinal and femoral orifices, potentially reducing the risk of unrecognized femoral hernias [2]. A recent registry-based study found comparable outcomes between open and MIS approaches for BGH, although the open group included both anterior and posterior repairs, limiting specific conclusions about the open posterior technique [42].

The advantages of posterior repair, including the lower incidence of early chronic postoperative pain observed in our study, may be explained by the dissection plane: both MIS and OPA avoid the inguinal canal and reduce nerve contact, unlike the anterior approach. Furthermore, while tacker fixation is common in MIS and may contribute to discomfort, OPA allows for more selective and potentially less traumatic fixation strategies, potentially lowering the risk of CPIP.

In our cohort, mesh fixation practices further illustrate these distinctions. MIS procedures (TAPP and TEP) showed heterogeneous fixation methods, including both absorbable and non-absorbable tackers, the latter being predominant and chosen at the surgeon’s discretion. This reflects ongoing debate in the literature regarding the superiority of any specific fixation method [43]. In contrast, OPA was uniformly associated with absorbable suture fixation, which may contribute to the lower incidence of early CPIP observed in our cohort.

Additionally, in our series, the longer operative time observed in the MIS group aligns with prior evidence identifying this as a drawback of laparoscopic hernia repair [39]. However, several factors may have contributed to the median operative time of nearly 120 min. These procedures were performed in the early phase of implementing laparoscopic techniques in the ambulatory setting, before the widespread use of barbed sutures for peritoneal closure for example. These factors likely prolonged operative time beyond what is currently achievable with more standardized workflows and contemporary instrumentation.

Nonetheless, no differences were observed in early postoperative complications, including SSOs. However, the use of non-absorbable skin sutures was significantly associated with superficial wound infection. While previous meta-analyses and RCTs report no consistent difference between absorbable and non-absorbable sutures [44, 45], our data suggest a possible trend favoring absorbable sutures. Nevertheless, the low number of infections limits the ability to draw firm conclusions, and larger series would be needed to validate it.

Our study also highlights a systematic difference in the type of anaesthesia used between groups, with OPA performed under spinal anaesthesia and MIS under general anaesthesia. We reported these differences but did not attribute independent clinical relevance to them, as current evidence suggests that both techniques are safe and effective, each with specific trade-offs [46–48]. Spinal anaesthesia has been associated with less immediate postoperative pain but higher risks of urinary retention and headache, whereas general anaesthesia allows shorter operating times and avoids some of the complications related to spinal techniques [46–48].

In the univariate analysis, younger age and recurrent hernias were both associated with a higher risk of early CPIP, in line with previous studies indicating that younger and recurrent patients may report more pain after hernia repair [49, 50]. However, multivariate logistic regression identified the surgical approach as the only independent predictor of early CPIP (AUC 0.84). This suggests that other variables, such as age or recurrence status, likely confounded the association, especially given their higher prevalence in the MIS group. Therefore, while patient-related factors may contribute to pain perception, the observed differences in early CPIP between groups appear to be more closely linked to the surgical technique itself. Importantly, the observed difference in pain outcomes between groups did not persist at long-term follow-up (> 12 months postoperatively).

Recurrence rates were similar between groups, with a median follow-up of 116 months (9.6 years). The only factor independently associated with CPIP at long-term follow-up was higher BMI (OR = 1.2, 95% CI: 1.024–1.405; *P* = 0.024), aligning with prior literature suggesting that both low and high BMI can increase postoperative chronic pain [51–53].

Overall, our results suggest that a purely OPA can offer comparable—or even superior—early postoperative pain outcomes compared to MIS, while maintaining equivalent safety and efficacy at long-term follow-up.

Nonetheless, there are inherent limitations that should be acknowledged. First, its retrospective design introduces potential selection and information biases, limiting causal inference. Because of the study design, we were also unable to systematically record preoperative inguinal pain, a known risk factor for CPIP, which limited our ability to adjust for it. Second, despite a high retention rate, some patients were lost to follow-up, particularly for subjective measures like pain. Third, surgical technique selection was not randomized but based on surgeon preference, introducing potential selection bias. Additionally, all procedures were performed by a single surgeon at a single centre, which may limit generalisability. The study population was also limited to patients selected for ambulatory surgery, excluding individuals with higher surgical risk or significant comorbidities, which may restrict the generalisability of the findings to broader patient populations. Furthermore, although structured telephone interviews were used to standardize long-term follow-up, this approach may have reduced sensitivity in detecting asymptomatic or subclinical recurrences compared with systematic in-person evaluations. Finally, relevant patient-reported outcomes—such as quality of life, satisfaction, and costs—were not systematically collected, which limits the ability to assess the full impact of each approach.

A key strength of the study is the long-term follow-up, with a median of over 9 years (range 6–14), which allows a robust evaluation of less frequent outcomes such as recurrence and CPIP. This duration of follow-up is rarely achieved in similar cohorts and adds weight to the reliability of our findings.

## Conclusion

Our findings suggest that OPA for BGH repair offers similar surgical outcomes comparable to those of MIS. It was associated with lower rates of early CPIP (3–12 months postoperatively), with no significant long-term differences in recurrence or CPIP. Although multivariate analysis identified the surgical approach as the main factor associated with early CPIP, other variables such as age, recurrence status, or fixation methods may also play a role. Higher BMI was the only independent predictor of CPIP at long-term follow-up. The extended follow-up duration enhances the reliability of our findings and supports the consideration of OPA as a viable, effective, and resource-efficient technique for repairing BGH in the ambulatory setting.

## Data Availability

Due to privacy and ethical restrictions, the data underlying this study cannot be made publicly available. Data may be shared by the corresponding author upon justified request and with permission from the Ethics Committee.
