# Correction to: Ambulatory bilateral groin hernia repair: open preperitoneal versus laparoscopic outcomes

**DOI:** 10.1007/s10029-025-03554-x

**Published:** 2026-01-07

**Authors:** Maria Jose Gomez-Jurado, Mireia Verdaguer-Tremolosa, Victor Rodrigues-Gonçalves, Pilar Martínez-López, María Martínez-López, Meritxell Pera, Mar Dalmau, Manuel López-Cano

**Affiliations:** 1 General and Digestive Surgery Department, Dr. Josep Trueta University Hospital, Girona, Spain; 2 General and Digestive Surgery Department, Vall d’Hebron University Hospital, Barcelona, Spain; 3 Department of Surgery, Universitat Autònoma de Barcelona, Barcelona, Spain; 4 Abdominal Wall Unit, General and Digestive Surgery Department, Vall d’Hebron University Hospital, Barcelona, Spain; 5 Institut d’Investigació Biomèdica de Girona (IDIBGI), Girona, Spain

**Correction to: Hernia (2026) 30:17**

10.1007/s10029-025-03523-4

In this article the affiliation ‘*Institut d’Investigació Biomèdica de Girona (IDIBGI)*,* Girona*,* Spain* ' for Author Maria Jose Gomez-Jurado was missing.

